# Use of Lithium in Pediatric Bipolar Disorders and Externalizing Childhood-related Disorders: A Systematic Review of Randomized Controlled Trials

**DOI:** 10.2174/1570159X21666230126153105

**Published:** 2023-05-12

**Authors:** Delfina Janiri, Lorenzo Moccia, Silvia Montanari, Valentina Zani, Claudia Prinari, Laura Monti, Daniela Chieffo, Marianna Mazza, Alessio Simonetti, Georgios D. Kotzalidis, Luigi Janiri

**Affiliations:** 1 Department of Geriatrics, Institute of Psychiatry and Psychology, Neuroscience and Orthopedics, Catholic University of the Sacred Heart, Largo Francesco Vito 1, Rome, 00168, Italy;; 2 Department of Psychiatry, Fondazione Policlinico Universitario Agostino Gemelli IRCCS, Largo Agostino Gemelli 1, Rome, 00168, Italy;; 3 UOS Clinical Psychology, Clinical Government, Fondazione Policlinico Universitario Agostino Gemelli IRCCS, Largo Agostino Gemelli 1, 00168, Rome, Italy;; 4 Centro Lucio Bini, Via Crescenzio 42, Rome, 00193, Italy;; 5 Menninger Department of Psychiatry and Behavioral Sciences, Baylor College of Medicine, 1 Baylor Plaza, Houston, 77030, TX, USA;; 6 NESMOS Department, La Sapienza, Faculty of Medicine and Psychology, Sant’Andrea University Hospital, University of Rome, Via di Grottarossa, 1035-1039, Rome, 00189, Italy

**Keywords:** Lithium, pediatric, bipolar disorder, conduct disorder, children, externalizing disorder

## Abstract

**Background:**

Lithium is the standard treatment for bipolar disorders (BD) in adults. There is a dearth of data on its use in the pediatric age. This review aimed to investigate the use of lithium in pediatric bipolar disorder (BD) and other externalizing childhood-related disorders.

**Methods:**

We applied the Preferred Reporting Items for Systematic Reviews and Meta-analyses criteria (PRISMA) to identify randomized controlled trials evaluating the use of lithium in pediatric (BD), conduct disorder (CD), attention deficit hyperactivity disorder, oppositional defiant disorder, and disruptive mood dysregulation disorder. The primary outcome of our study was to evaluate the efficacy of lithium compared to a placebo or other pharmacological agents. The secondary outcomes were acceptability and tolerability.

**Results:**

Twelve studies were eligible, 8 on BD and 4 on CD. Overall, 857 patients were treated with lithium. No studies for externalizing disorder diagnoses were identified. Regarding BD patients (n = 673), efficacy results suggested that lithium was superior to placebo in manic/mixed episodes but inferior to antipsychotics. Lithium efficacy ranged from 32% to 82.4%. Results on maintenance need to be expanded. Comorbidity rates with other externalizing disorders were extremely high, up to 98.6%. Results in CD patients (n= 184) suggested the efficacy of lithium, especially for aggressive behaviors. No severe adverse events directly related to lithium were reported in BD and CD; common side effects were similar to adults.

**Conclusion:**

This systematic review supports the use of lithium in BD and CD as an efficacious and generally well‐tolerated treatment in the pediatric age. However, evidence is limited due to the paucity of available data.

## 1 INTRODUCTION

Lithium is the standard treatment for bipolar disorders (BD) [1, 2], and one of the most consistently effective pharmacological treatments in psychiatry [3]. The FDA approved lithium for adult acute mania in 1970 and the prevention of recurrences in adult BD in 1974. Even though the onset of BD typically peaks around 15-25 years of age [4], lithium use has not been extended to youths aged 12-17 until the early 21^st^ century, mostly on the basis of adult trials. The FDA prompted child psychiatrists to collect evidence for lithium in childhood/adolescent BD, a still ongoing process.

BD in pediatric age has an average prevalence of 3.9%, depending on the use of broad or restrictive diagnostic criteria [5]. When diagnostic criteria (DSM/ICD) are more loosely interpreted, pediatric BD may include clinical features shared with other externalizing childhood-related disorders, which are characterized by a tendency to experience distress outwards and behavioral problems [6]. In particular, conduct disorder (CD), attention deficit hyperactivity disorder (ADHD), oppositional defiant disorder (ODD), and disruptive mood dysregulation disorder (DMDD) may often be considered in children as differential diagnoses of BD [7]. They may share mood/emotional dysregulation symptoms, including mood alterations, irritability, hyperarousal and increased reactivity to emotional stimuli [7]. Furthermore, they are frequent comorbid conditions in youths with BD [8-11]. Based on these observations, the use of lithium in pediatric age has been extended in the clinical practice to these additional specific disorders [12-16]. This use is based on the hypothesis that lithium may impact not only mood episodes but also specific psychopathological dimensions influenced by mood/emotional dysregulation [14].

A recent systematic review reported data on the efficacy and tolerability of lithium for the treatment of acute mania in children with BD [17]. With respect to previous literature overviews [18], the authors found new evidence for the use of lithium in pediatric mania, highlighting the dearth of available data. Nevertheless, they did not provide information on the efficacy of lithium on depressive symptoms and the long-term outcomes of BD. Regarding the use of lithium in other externalizing childhood-related disorders, no study to date summarized the available evidence on drug efficacy and acceptability/tolerability. The aim of this systematic review is to fill this gap, focusing on randomized controlled trials evaluating the use of lithium in pediatric BD, CD, ADHD, ODD and DMDD.

## 2 METHODS

### 2.1 Search

We applied the Preferred Reporting Items for Systematic Reviews and Meta-analyses criteria (http://www.prisma-statement.org/) to identify randomized controlled trials using lithium in pediatric Bipolar Disorder (BD), Conduct Disorder (CD), Attention Deficit Hyperactivity Disorder (ADHD), Oppositional Defiant Disorder (ODD) and Disruptive Mood Dysregulation Disorder (DMDD).

Studies were eligible if they compared lithium with a placebo or an alternative active drug. Lithium could be administered by any method at any dose. Studies were not excluded based on the risk of bias, but potential biases were highlighted and discussed in the current review. Details of the search and article eligibility criteria, as well as the databases used can be found in the Supplement. Eligibility was established with consensus among authors through Delphi rounds.

### 2.2 Primary and Secondary Outcomes

The primary outcome of our study was to evaluate the efficacy of lithium in pediatric age defined by increase/ decrease in scores on any validated rating scale from baseline and endpoints, between individuals treated with lithium and individuals treated with placebo or other pharmacological agents. As secondary outcomes, we also considered acceptability and tolerability. Acceptability was defined by two measures: (i) the difference in discontinuation rates for any reason between lithium and the control group, (ii) the difference in discontinuation rates for adverse events due to lithium exposure. Tolerability was defined as a difference in adverse events between lithium and control groups.

### 2.3 Data Extraction

Specific data of the eligible full-version articles were carefully extracted and filled into the developed extraction form. The extracted outcomes, when available for each eligible study, consisted of the following:

1) Data on efficacy, tolerability and acceptability. For efficacy outcomes, response rates were reported if available.

2) Demographic characteristics.

3) Clinical characteristics of included patients (diagnosis, duration of illness, illness onset, CD, ADHD, ODD, and DMDD comorbidity; concomitant drug treatment, recruitment setting).

4) Design of the study including information on blindness.

5) Conclusions and limitations (when reported in the original study).

The risk of bias was assessed using the Cochrane Risk of Bias Tool 2 (RoB2) [19]. Inconsistencies in applying the tool were discussed among all authors. After discussion, a full agreement was reached. Results were reported according to the RoB2 recommendations [19]. The RoB for all eligible studies is shown in Fig. (**1**).

## 3 RESULTS

At end of the eligibility process, we included 12 independent trials, for a total of 857 patients. All the included studies were randomized controlled trials, written in English, although this was not a prerequisite. Further information on the results of the search can be found in the Supplement. The results of our search are shown as a PRISMA flowchart in Supplementary Fig. (**S1**) with the reasons for exclusion.

A description of the included studies, including information on the study population, study design, efficacy, acceptability, tolerability, conclusions and limitations, can be found in Table **1**.

Based on the results, we decided to divide the studies into two groups: those focusing on BD and those focusing on CD, since the other externalizing childhood-related disorders (ADHD, ODD, DMDD) produced no eligible results. Based on this subdivision, 8 studies emerged from our search in the “bipolar disorder” category [20-27] and 4 in the “conduct disorder” [13, 16, 28, 29] category, as described below. The demographic and clinical characteristics of patients included in the eligible articles are shown in Table **2**.

### 3.1 Studies on Bipolar Disorder

The 8 included studies involved a total of 673 patients, 346 of whom were males and 327 females, mostly outpatients, with a 5-18 years age range. Most of the studies reported data from small sample sizes (<100 individuals); the larger study was by Geller *et al.* [25] including 279 outpatients. The studies involved patients with diagnoses of Type I or II BD, mostly referring to the DSM-IV criteria, with ongoing manic or mixed episodes. Only Findling *et al.* [24] and Kafantaris *et al.* [22] included patients that were already undergoing pharmacological stabilization, as they were maintenance/discontinuation trials. Geller *et al.* [20] included patients who already had a diagnosis of BD, but this was comorbid with substance use disorder. Of note, no randomized controlled trials regarding lithium use in adolescents during the depressive phase could be found. Most of the studies reported data from outpatients. Comorbidity rates with other externalizing disorders were extremely high, ranging between 47.1% to 92.8% for ADHD, 38% to 90% for ODD, 7% to 15% for CD, and 20% to 98.6% for DMDD.

Five of these studies were randomized, double blind controlled trials (*vs*. placebo, *vs*. divalproex sodium or *vs*. quetiapine) (Table **1**); the Treatment of Early Age Mania Study (TEAM) study was single blind and compared risperidone and divalproex [25]; the remaining two were open, non-blind studies; one [21] comparing lithium, divalproex, and carbamazepine; the other [23] lithium or divalproex in combination with risperidone. All independent studies were randomized to two or three parallel groups.

Regarding the duration of the studies, 6 studies lasted 6-8 weeks (Table **1**); two studies deviated significantly from this, Pavuluri *et al.* [23] with a treatment duration of 6 months, and Findling *et al.* [24], a maintenance study, which lasted 76 weeks.

Two of the included studies were parts of the TEAM and The Collaborative Lithium Trials (CoLT). Regarding the TEAM study, the study that was considered as the primary study was Geller *et al.* [25], where drug-naïve patients (for anti-manic drugs) were randomized to three parallel groups (lithium, divalproex, risperidone); patients who did not respond to this phase of the study together with those who were not-drug naïve at the beginning, were then included in the next phase of the study (cross taper/add on) described in Walkup *et al.* [30]. This study was therefore considered an additional TEAM because it is not possible to determine which patients overlap with those of the Geller study [25]. The other two studies included additionally are Salpekar *et al.* [31] and Vitiello *et al.* [32], both *post hoc* analyses of the same patients of Geller *et al.* [25], investigated other specific areas (the first reduction of suicidality, the second treatment moderators and predictors of outcome). The additional TEAM studies are reported in Supplementary Table **S1**. Regarding the CoLT study, the study that was considered the primary study was by Findling *et al.* [26], which was a randomized, double blind controlled trial. Two other papers that were parts of the CoLT study were excluded because they did not compare lithium with a placebo or an alternative active drug. Another study reported data on maintenance and discontinuation due to mood symptoms but considered a smaller sample size [33]. The additional CoLT studies are summarized in Supplementary Table **S1**.

#### 3.1.1 Efficacy

From the analysis of the results concerning the efficacy in BD, what emerges is the superiority of lithium over placebo, but also the greater efficacy over lithium itself of risperidone and quetiapine during the manic phase. No differences were found between divalproex and lithium.

Only two studies compared lithium with placebo [20, 26]. In Findling *et al.* [26] the response rate was 32% for the lithium group *vs*. 21% for placebo. In patients with comorbid substance use and BD [20], lithium was found to be significantly more effective than placebo, with response rates of 46.2% *vs*. 8.3%. Other response rates of lithium were 35.6% in the TEAM study [25], 38% in Kowatch *et al.* [21], 82.4% in Pavuluri *et al.* (where lithium was combined with risperidone) [23] and 49% in Patino *et al.* [27]. In the studies by Findling *et al.* [24] and Kafantaris *et al.* [22], overall, there were no significant effects of treatment on the target outcome considered, namely exacerbation rates of mood symptoms after stabilization.

To compare the results obtained, it is necessary to look at the endpoints chosen, and the efficacy measures used (Table **1**). In this case, although not completely uniform, the studies appear to be sufficiently comparable in that four studies used at least a 50% decrease in the Young Mania Rating Scale (YMRS) scores as a measure of response; in addition, two of them combined other scales, *i.e*., the Clinical Global Impressions-Improvement (CGI-I) [24] or CGI-Severity, Children’s Depression Rating Scale-Revised (CDRS-R) and the Children’s Global Assessment Scale (CGAS) [20, 24], while only the TEAM study [25] chose a different scale for the definition of mania, the K-SADS (Kiddie Schedule for Affective Disorders and Schizophrenia) Mania Rating Scale (KMRS), and a different definition of response to treatment, *i.e*., the Clinical Global Impressions for Bipolar Illness Improvement Mania; CGI-BP-IM score of 1 or 2.

#### 3.1.2 Acceptability

Discontinuation rates for any reason.

Lithium discontinuation rates were not significantly higher than placebo according to Findling *et al.* (30% *vs.* 25%, respectively) [26], while there was a significant difference in the Geller *et al.* study [20] (23% lithium *vs.* 8.3% placebo). In contrast, lithium discontinuation rates were higher compared with risperidone in the TEAM study (32.2% *vs.* 15.7%) and compared with divalproex sodium (32.2% *vs.* 26.0%) [25]. In the Kowatch *et al.* study [21], discontinuation rates were similar among all groups, 15% for lithium and carbamazepine and 13% for divalproex. Likewise, the Pavuluri *et al.* study [23] reported no difference in discontinuation rates between the two groups. The only study comparing lithium with quetiapine [27] showed a higher discontinuation rate for lithium compared with quetiapine (41% *vs*. 21% respectively). The percentages presented so far, however, are related to drop out for all causes and therefore do not reflect in the most exact way the acceptability of the drug administered, as they include, for example, patients who have left the studies because they withdrew consent, or moved to another state, or because they were arrested. For this reason, we tried to calculate the most likely percentages of acceptability by selecting, for each study, only the causes of drop out attributable to the administered treatments, such as discontinuation due to severe adverse effects and lack of effect or physician’s decision.

According to this paradigm, discontinuation rates were 20% for lithium *vs*. 14% for placebo [26], while Geller *et al.* [20] did not report data on the causes of dropout; lithium 14% *vs*. risperidone 6% *vs.* divalproex 9% (TEAM study) [25]; lithium plus risperidone 10% *vs*. divalproex plus risperidone 0% [23]; lithium 35% *vs*. quetiapine 19% [27]; lithium 6.7% *vs*. DVPX 10% [24]. It was not possible to calculate these acceptability rates for the Kowatch *et al.* [21] and the Kafantaris *et al.* study [22] because the causes of dropout were not reported.

#### 3.1.3 Tolerability

For tolerability we focused on the most common Adverse Events (AEs) and severe Adverse Events (SAEs). It has not been possible to compare side effects characteristics due to the heterogeneity of assessment tools. When reported in the original studies, frequencies of the most common SAEs are reported in Table **1**. Specifically, compared to placebo, lithium displayed more SAEs both in Geller *et al.* [20] and Findling *et al.* [26], the latter with 9.4% for the lithium group *vs*. 7.1% for placebo, although the authors specified that none of these events were believed to be related to the study medication; similarly, none of the five SAEs found in the TEAM study were deemed to be related to the randomized medication by the principal investigators [25]. All other studies did not report severe AEs. The most common AEs for each trial are described in Table **1**.

### 3.2 Studies on Conduct Disorder

In this group, 4 studies were included, for a total of 184 patients, 150 of whom were males and 34 females, aged between 3 and 18 years. All the studies reported data from small sample sizes (<100 individuals). These studies differ from those previously reported by the diagnostic references used, as they refer to DSM-III or DSM-III-R. It means that attention should be paid to the definition of this disorder, whose diagnostic criteria have changed significantly over the years and between different editions of the DSM (*i.e*. “In DSM-III the category of conduct disorders included cases in which there is a repetitive and persistent pattern of aggressive or nonaggressive conduct that violates either the rights of others or major age-appropriate societal norms or rules”). All patients included in the studies were hospitalized.

Three studies were randomized double blind controlled trials [13, 16, 28], one study [29] was a single-blind placebo-controlled trial for patients with conduct disorder (CD) and ≥3 aggression episodes during one week of hospitalization. Three studies compared lithium with a placebo while one conducted a parallel comparison of lithium with a placebo or haloperidol (Table **1**).

Regarding the duration of the studies, Rifkin *et al.* [29] lasted only two weeks; Campbell *et al.* [28] and Malone *et al.* [13] lasted 4 and 6 weeks, respectively. The Campbell *et al.*’s study [16] was the longer study available, with a treatment duration of 10 weeks.

#### 3.2.1 Efficacy

With the exception of one study, efficacy results indicated that lithium was generally superior to a placebo, or at least as effective as haloperidol, in children and adolescents with CD, especially as regards the reduction of aggressive and hostile behaviors. In Malone *et al.* [13] response rate was 80% for lithium and 30% for placebo (*p* = 0.04); in Campbell *et al.* [16], the response rate was 68% for lithium and 40% for placebo (*p* = 0.003). In contrast, in Rifkin *et al.* [29] the advantage of lithium (response rate of 21.4%) over placebo (response rate of 8.3%) was not significant. However, the latter study was of shorter duration compared to the other two (2 weeks of treatment). In the three-arm comparison [28], both lithium and haloperidol were superior to the placebo, but no differences were found between the two drugs. The primary endpoints of included studies all pertained to aggressive behaviors; two studies used the Overt Aggression Scale (OAS) [13, 29] and the other two used aggression factors derived from the Children's Psychiatric Rating Scale (CPRS) [16, 28].

#### 3.2.2 Acceptability and Tolerability

In the four eligible studies, no drop-out rates were reported for lithium. A similar dearth of data regarded AEs, with SAEs not reported in any study and mild-to-moderate AEs were reported for all treatment groups compared with placebo, with gastrointestinal problems, tremors, polydipsia/polyuria, and headache/dizziness being the most common for lithium-treated patients.

#### 3.2.3 Risk of Bias

Risk of bias results according to RoB2 are reported in Fig. (**1**).

## 4 DISCUSSION

**Fig. (1):**
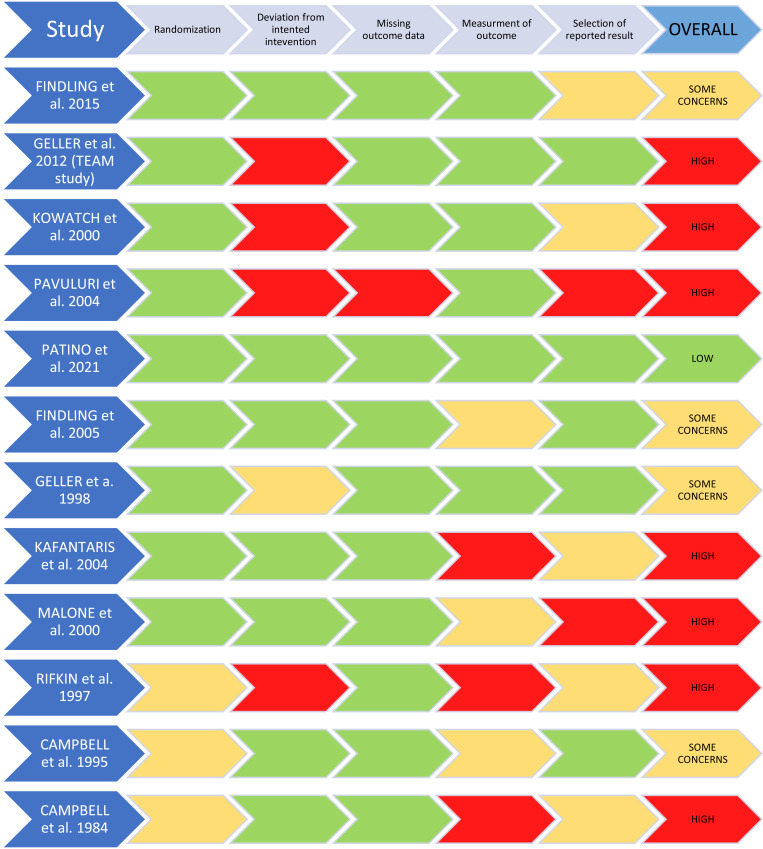
Risk of bias of included studies according to the Cochrane tool.

To the best of our knowledge, this is the first systematic review targeting the efficacy, acceptability, and tolerability of lithium *versus* placebo or other active comparator drugs in children and adolescents with BD and other externalizing childhood-related disorders, including CD, ADHD, ODD, and DMDD. One of the main findings from our review is the lack of available evidence; reviewed studies are few, heterogeneous and presented methodological limitations that restricted the generalizability of the results (Tables **1**, **2** and Fig. **1**).

### 4.1 Longitudinal Controlled Studies

Eight longitudinal controlled trials involving a total of 676 pediatric patients with BD were included in the review. Among those studies, six assessed the efficacy and tolerability of lithium compared to either placebo [20, 26], divalproex [21, 23, 25], risperidone [25], carbamazepine [21], or quetiapine [27]. Only two studies compared lithium to placebo [20, 26] and showed the superiority of lithium, in line with the adult population [34, 35]. Findling *et al.* [26] found lithium to be superior to placebo in pediatric participants with BD-I/manic or mixed episodes. Geller *et al.* [20] assessed BD patients with secondary substance abuse and observed that individuals treated with lithium showed fewer mood symptoms and lower rates of substance use relapse than the placebo group. The authors included patients with a mean age of 16.5 years [20] and 9.5 years [26], respectively. Keeping in mind the very limited number of observations, results suggest that lithium may be effective in different age groups. Further studies are needed to confirm these initial observations and to clarify whether age modifies the effect of lithium. The studies comparing lithium to other pharmacological comparators provided mixed evidence, especially in adolescent patients with an ongoing acute manic episode. The largest study that assessed the anti-manic effects of lithium (part of the TEAM study [25]), compared it to either divalproex or risperidone, found lithium and divalproex less effective than risperidone in treating mania. Similarly, the study of Patino *et al.* [27] showed that quetiapine was associated with a statistically significant greater reduction in manic symptoms compared to lithium, though treatment with both lithium and quetiapine led to clinical improvement. Taken together, these findings suggest that acute mania in children with BD may preferentially respond to antipsychotics rather than to lithium or anticonvulsants. Results are not surprising and in line with robust evidences in adults, showing that antipsychotic drugs were more effective than mood stabilizers in treating mania in the short-term [34]. Findings may rely on the intrinsic pharmacological efficacy of antipsychotics in treating manic symptoms, in particular, psychotic symptoms and psychomotor agitation [36]. However, most of the included studies in the current review involved patients with a very early BD onset and a clinical presentation including prominent mixed features as well as high rates of comorbid ADHD (up to 92.8%) and concurrent stimulant use, which may predict poorer response to lithium treatment [37, 38]. In addition, the mean length of a manic episodes in the study with the larger sample size [25] was almost 5 years, possibly indicating an absence of episodic mood disturbances that may be more likely to respond to lithium. Moreover, the very short follow-up period may underestimate the potential long-term benefit of lithium.

### 4.2 Maintenance and Discontinuation Studies

The above-presented studies apply only to the acute manic/mixed phase of BD and do not inform the clinically important issues of relapse prevention and mood stabilization in the medium and long term. Only two studies in the current review included patients that were already undergoing pharmacological stabilization, as they were maintenance/discontinuation trials. Findling *et al.* [24] compared time to mood relapse in patients treated with lithium or divalproex, while Kafantaris *et al.* [22] conducted a placebo-controlled trial. Both studies did not find any difference between lithium and placebo [22] or divalproex [24]. However, these studies present methodological flaws (Fig. **1**), including a short observation period for mood stabilization to show off [22] and small sample sizes [22, 24]. Conversely, in one of the additional CoLT studies (Supplementary Table **S1**), Findling *et al.* [33] found lithium to be superior to placebo in the maintenance treatment of BD for 28 weeks. These results are in line with a recent naturalistic longitudinal study involving 413 youths with BD, aged from 7 to 17 years [39]. The authors followed up patients for 10 years and found that lithium treatment was associated with greater control of mood symptoms and relapses compared to other mood-stabilizing medications. Results strongly suggest the need for longer follow-up observation time in maintenance randomized controlled trials in pediatric age. Interestingly, the same study also found in the lithium group less depressive symptoms, suicide attempts, and psychosocial impairment, as well as reduced aggressive behavior [40]. Based on the convincingly proven prophylactic activity of lithium in affective disorder in adulthood [41, 42], further longitudinal controlled clinical trials with larger samples and longer follow-up are needed to confirm the efficacy of lithium in children and adolescents with BD, especially as regards the prevention of suicide and mood episodes relapse.

### 4.3 Adverse Events and Tolerability

The tolerability profile for lithium in the short-term was generally good across the reviewed studies, with few severe AEs and most common side effects including nausea, headache, and increased urination, all of which are usually related to plasma lithium concentration [43]. Target serum concentrations across studies ranged from 0.5 to 1.3 mEq/L, with the latter in the dangerous adult range. Common side effects in lithium‐treated pediatric patients are similar to those observed in adults and usually show a dose-response pattern [43]. Furthermore, there is evidence that multiple daily dose regimens, which result in lower peaks in plasma lithium levels, as well as slower titration, may minimize the risk of AEs [44]. Of note, a recent pharmacokinetic study conducted in 61 pediatric patients with BD demonstrated that a daily lithium carbonate dose of 25 mg/kg total body weight in two doses/day, resulting in a mean lithium plasma level of approximately 0.7 mEq/L, was associated with a 50% reduction in manic symptoms in 74% of patients over a 24‐week follow-up period [45]. However, it should be mentioned that some adverse events observed in adult populations include leukocytosis, hypothyroidism and renal tubular dysfunction [46]; since adverse events in adult populations do not differ from those in pediatric age populations, special efforts should be made to address them in young patients.

Four studies involving a total of 184 patients evaluated lithium treatment in CD [13, 16, 28, 29]. These studies spanned from 1984 to 2000, while other studies were published between 1998 and 2021, thus the former was considerably older than the latter. Included studies assessed the efficacy and tolerability of lithium compared to placebo or placebo and haloperidol (Table **1**). With the exception of the study of Rifkin *et al.* [29], reviewed studies indicated that lithium was generally superior to placebo, or at least as effective as haloperidol, in children and adolescents with CD, especially as regards the reduction of aggressive and hostile behaviors. Furthermore, the study of Rifkin *et al.* [29] had a 2-week treatment duration, which was probably insufficient for the therapeutic effect of lithium to show off, and was the only single-blind study included in the CD category. The major limitations of included studies were that only inpatients were enrolled (Table **2**), and the mean length of follow-up was 4 ± 2 weeks (Table **1**). Accordingly, it is possible that the response of lithium may have not appeared after so short period or did not continue after discharge from the hospital to other clinical settings. Furthermore, results from the risk of bias assessment caused concerns for these studies (Fig. **1**). Keeping in mind these limitations and the limited number of observations, the findings provide initial evidence that lithium may be beneficial in the short-term treatment of aggression in children and adolescent inpatients with CD.

Lithium tolerability appeared to be acceptable across reviewed studies in CD and no SAEs were reported. The most common side effects in lithium-treated patients included nausea, headache, and polyuria. In the only study comparing lithium to another drug comparator (*i.e.,* haloperidol) [28], no significant differences between the two drugs were observed regarding the average number of side effects, though side effects attributable to haloperidol seem to interfere with patients’ functioning more than lithium, as indicated by the Clinical Global Impression ratings.

### 4.4 Comorbidity Issues

In the current review, we found no studies focusing on the use of lithium in ADHD, ODD and DMDD specifically. Nevertheless, in the BD category, we found very high comorbidity rates for these other externalizing disorders, ranging between 47.1% to 92.8% for ADHD, 38% to 90% for ODD, and 20% to 98.6% for DMDD (Table **2**). These results are in line with previous findings, showing that externalizing disorders are often comorbid conditions in pediatric age. Results may also suggest, despite the lack of direct evidence, a potential for using lithium in ADHD, ODD, and DMDD. Lithium may impact not only those symptoms, such as elation and increased motor activity, that more closely suggest narrowly defined manic‐depressive illness, but also specific psychopathological dimensions shared with other externalizing disorders. In particular, it may mitigate mood/emotional dysregulation symptoms [47], including mood swings, irritability, hyperarousal and increased reactivity to emotional stimuli. In line with this, lithium exposure was found to be associated with reduced emotional dysregulation among adolescents with BD [48]. Furthermore, it may improve cognitive outcomes of ADHD patients, as it showed to improve cognition in pediatric BD, although the effect was small [49]. Further targeted studies are warranted to confirm these initial speculations.

### 4.5 Limitations

Before presenting our conclusions, we must acknowledge some limitations which reduce the generalizability of our results. First, the small number of included studies prompts caution in interpreting results. This limitation mainly relies on the dearth of available literature on the use of lithium in the pediatric age. Second, despite we included only patients with a DSM-based diagnosis, patient samples were extremely heterogeneous. This concerns age ranges, duration of illness, comorbidity rates, concomitant medications, treatment duration, and recruitment setting (Table **2**). Third, in the BD section, most included studies involved patients with high rates of comorbid externalizing disorders, making it difficult to disentangle the efficacy of lithium in each of the specific diagnostic entities. Furthermore, most studies used a short outcome, and this might have missed the known long-term beneficial effects of lithium. Open-label studies are more likely to detect changes, but of the few open-label studies included here, one used a 6-week endpoint [21] and two using 20-week and 6-month endpoints had small samples [23, 24] (Table **1**). One open-label naturalistic study that observed 90 patients on lithium for at least 6 months found a 60% response [50], which was higher than most studies included here. This study compared lithium to valproate and antipsychotics. Another open-label study that did not use controls, found a 68.3% response and 53.7% remission in 41 patients at the 16-week endpoint [51]. Knowing that lithium is a slowly acting drug, longer follow-ups are needed to obtain stronger results. Another issue is suicidal ideation and attempts. It is supported that lithium reduces suicide rates in patients who use it [52-54]. In our study, the one study that focused on suicidal ideation and attempts [31], did not find treatments to affect suicidal ratings; furthermore, overall occurrence of suicidal ideation and attempts was infrequent.

## CONCLUSION

In conclusion, the results of this systematic review support the use of lithium as an efficacious and generally well‐tolerated treatment in pediatric age; however, evidence is limited in that available data are few. Specifically, BD efficacy results suggested that lithium was superior to placebo in manic/mixed episodes but inferior to antipsychotics. However, this can affect the study’s observation length, as acute manic episode studies usually last 3 weeks, and this is an adequate time to observe the effects of incisive antipsychotic agents but may be too short for observing the stabilizing effects of lithium. Findings on the effect of lithium in maintaining mood stabilization need to be expanded. Further controlled l trials in pediatric populations with larger samples and longer follow-up times are needed to confirm the well-known clinical practice-based observation, that lithium is effective in preventing relapses of mood episodes. Further studies are also required to specifically investigate the use of lithium in the prevention of suicide risk in youth, something that has been convincingly shown in adults [55]. The results of our study also suggest a potential use of lithium in CD, in particular in modulating aggressive behaviors. Future trials are required to confirm these initial findings and to investigate lithium use in other externalizing childhood-related disorders, *i.e*. ADHD, ODD and DMDD. Finally, there is a need for studies to include samples with increased clinical homogeneity and lower comorbidity rates.

## Figures and Tables

**Table 1:** Longitudinal controlled clinical trials using lithium in pediatric populations.

**Study**	**Patients**	**Design**	**Results**	**Discontinuation Rates ** **(Acceptability)**	**Tolerability/** **Adverse Effects**	**Conclusions**	**Limitations**
**Bipolar Disorder**							
Geller *et al*., 1998 [20]	USA N = 25 pts (12-18 yr, M:16, F:9) with DSM-III-R SDD and BD, randomized: - Li^+^: N = 13; - Pla: N = 12.	6-wk, randomized, DB, Pla-controlled, trial. Primary outcome: effectiveness of Li^+^. - Li^+^ dose: BL dose: 600 mg/d; daily dose: 1.769 mg. - Psychometric assessment: **CGAS> = 65**; K-SADS, SDD template, ADI, LSES. Also: random urine samples.	RR (CGAS) Li^+^: 46.2% Pla: 8.3% Lithium superior to placebo (*p* = .046) Random urine samples resulted positive after wk 3 are significantly less with Li^+^.	All reasons: Li^+^: 23% Pla: 8.3% Attributable to treatments: not calculable because of the lack of described reasons.	SAEs/AEs Significant differences on SAE were found between the Li^+^ and Pla groups for thirst, polyuria, nausea, vomiting, and dizziness. Polyuria and polydipsia occurred with Li^+^.	Li^+^ reduced both SDD and BD in this trial, and it was well tolerated.	Small sample size. No follow up.
Kowatch *et al*., 2000 [21]	USA N = 42 outpts (6-18 yr; M:26, F:16) with DSM-IV BD-I or II, acute phase of mixed or manic episode, randomized: - Li^+^: N = 13 - CBZ: N = 13 - DVPX: N = 15.	6-wk, randomized, open, trial. Primary outcome: Reduction in BL YMRS score ≥ 50% - Li^+^ dose: at BL 30 mg/kg/day level: 0.88 ± 0.35 mEq/L. - Psychometric assessment: **YMRS**, CGI-BP.	RR (YMRS): Li^+^ 38%, DVPX 53% CBZ 38% Change from baseline Cohen’s *d* (YMRS): Li^+^:1.06; carbamazepine: 1.00; DVPX: 1.63 No significant difference between the 3 groups in response.	All reasons: Li^+^ = 15% CBZ = 15% DVPX = 13% Attributable to treatments: not calculable because of the lack of described reasons.	No SAEs. Most common AEs - Li^+^: nausea (21%) and ↑ appetite (14%); - CBZ: nausea (46%), sedation (15%); - DVPX: nausea (20%) and sedation (20%).	There were large effect sizes for all 3 treatments. No significant difference between the 3 groups in response	Small sample size. Lack of Pla control. Open design.
Kafantaris *et al*., 2004 [22] (phase II)	USA N = 40 inpts (12-18 yr; M:21, F:19) with DSM-IV BD-I, responders to previous 4-wk treatment with Li^+^, randomized: - Li^+^: N = 19; - Pla: N = 21.	2-wk, randomized, DB, Pla-controlled, maintenance trial. Primary outcome: exacerbation rates of mood symptoms. - Li^+^: serum level 0.99 mEq/L. - 3 days Li^+^ taper period. - Psychometric assessment: **CGJ**, YMRS; Ham-D; BPRS; CGI; CGAS	RR Exacerbation rates (by GCJ): 52.6% with Li^+^, 61.9% with Pla. No significant difference in exacerbation rates	Not reported	No SAEs. Most common AEs: 67.5% had some side effects causing mild impairment. diarrhea and ↑ appetite.	No significant difference in exacerbation rates between those continuing Li^+^ and those switched to Pla.	Short stabilization period. Potential withdrawal syndrome after Li^+^ discontinuation. Difference in residual symptoms between the groups. Mostly inpts sample. Expectation of worsening by the research team.
Pavuluri *et al*., 2004 [23]	USA N = 37 outpts (5-18 y; M:26, F:16) with DSM IV BD-I mixed or manic episode, YMRS > 20 at BL, randomized: -Li^+^ and Risp: N: 17; -DVPX and Risp: N: 20.	6-mo, randomized, open trial. Primary outcome: Reduction in BL YMRS score ≥ 50% - Li^+^/Risp dose: 0.9 mEq/L + Risp 0.75 ± 0.75 mg; - DVPX/Risp dose: 106 μg/dL Risp: 0.70 ± 0.67 mg. - Psychometric assessment: **YMRS**; CGI-BP-Improvement subscale of <2 and >51 CGAS score.	RR (YMRS) Li^+^/ Risp group: 82.4% DVPX/Risp group: 80%. RR (CGI-BP-Improvement +YMRS) Li^+^/Risp group: 70.6%, DVPX+Risp group: 70.0%. RR ((CGI-BP-Improvement +YMRS+CGAS): Li^+^/Risp group: 64.7% DVPX+Risp group: 60.0% No significant difference between groups	All reasons: Li^+^/ Risp: none (among patients included in the analyses). DVPX/Risp: none. Attributable to treatments: Li^+^/Risp: none (among patients included in the analyses). DVPX/Risp: none.	No SAEs. Most common AEs. - Li^+^/Risp: weight gain (35%), sedation (24%), nausea (24%), ↑ appetite (24%), stomach pain (24%). - DVPX/Risp: weight gain (40%), sedation (20%), ↑ appetite (20%).	Both DVPX/Risp and Li^+^/Risp show strong effects. Both combination treatments were well tolerated. No significant difference between groups	Sample size was not sufficient to show between-group differences on effect sizes or on efficacy measures. Open, single-site trial where unintentional introduction of bias was a possibility.
Findling *et al*., 2005 [24]	USA N = 60 outpts (5-17 yr; M:39, F:21) with BD-I or II according to DSM-IV, stabilized with DVPX+Li^+^ (4 wks of symptom remission, CDRS-R ≤ 40, YMRS ≤ 12.5 and CGAS ≥ 51), randomized: - Li^+^: N = 30; - DVPX: N = 30	76-wk, randomized, DB, maintenance trial (after phase I open-label combination Li^+^/ DVPX for up to 20 wks). Primary outcome: time to mood relapse - Li^+^ dose: serum level 0.84 mmol/L. - DVPX dose: between 50 and 100 mg/mL. - Psychometric assessment: K-SADS; CGI-I; CGI-S; **CGAS**; **YMRS**; **CDRS-R**.	Time to mood relapse did not differ between the Li^+^ and DVPX treatment groups.	All reasons: not reported. Attributable to treatments: DVPX: 10% Li^+^: 6.7%	No SAEs. Most common AEs: - Li^+^: emesis (30.0%), enuresis (30%); - DVPX: headache (23.3%), stomach pain (23.3%). 75% of pts reported side effects after randomization.	Time to mood relapse did not differ between the Li^+^ and DVPX treatment groups.	Small sample. No Pla-controlled.
Geller *et al*., 2012 Team study [25]	USA N = 279 outpts (6-15 y; M:139, F:140) with DSM-IV BD-I, mixed or manic episode for at least 4 wks preceding BL, with CGAS ≤ 60, randomized: - Li^+^: N = 90 - Risp: N = 89 - DVPX: N = 100	8-wk, randomized, single blind trial. Primary outcome: **CGI-BP-IM** score of 1 or 2 Secondary outcome: KMRS. - Li^+^ dose: 1.1 to 1.3 mEq/L - DVPX dose: 111 to 125 μg/mL - Risp dose:4 to 6 mg/d. - Psychometric assessment: **CGI-BP-IM**; CGAS.	RR (**CGI-BP-IM)**: 68.5% for Risp 35.6% for Li^+^ 24.0% for DVPX. Higher response risperidone *vs.* lithium (*P* <.001) and *vs.* divalproex (*P* <.001). Response to lithium *vs.* divalproex sodium did not differ.	All reasons: Li^+^: 32.2% Risp: 15.7% DVPX: 26.0% Attributable to treatments: Li^+^ 14% Risp: 6% DVPX: 9%	SAEs 5 SAEs but “none were deemed related to randomized medication by the principal investigators.” Most common AE* - Li^+^: Nausea (33%), vomiting (20%), dry mouth (20%), excessive thirst (41%), frequent urination (27%); - Risp: Weight gain (96%), nausea (17%), fever (10%), weight loss (10%), dry mouth (10%); - DVPX: Nausea (23%), vomiting (11%), weight gain (71%), diarrhoea (17%) fever (9%). *Identified by calculating the percent change from BL to EP by Duffy *et al*.	Risp was significantly superior to Li^+^ and DVPX, but adverse effects raise concern for long-term treatment.	No valid diagnostic biological measure for childhood BP disorder. Uncertain validity of preschool diagnoses. Too few nonpsychotic subjects for meaningful analyses of this subgroup.
Findling *et al*., 2015 (part of CoLT study) [26]	USA N = 81 outpts (7-17 yr; M:37, F:44) with DSM-IV BD-I, mixed or manic episodes, 2:1 randomized: - Li^+^: N = 53 - Pla: N = 28	8-wk, randomized, DB, Pla-controlled trial. Primary outcome: reduction in BL YMRS score ≥50% and a CGI-I score of 1 or 2. - Li^+^ dose: 600 or 900 mg/d; dose 1483 mg/d serum level 0.98 ± 0.47 mEq/l. - Psychometric assessment: **YMRS**, CDRS-R, CGI-S, CGAS, C-SSRS.	RR (YMRS): 32% Li^+^ 21% Pla Lithium superior to placebo (*p* = 0.03); Cohen’s d (YMRS): 0.53 (95% CI: 0.06 to 0.99).	All reasons: Li^+^: 30% Pla: 25% Attributable to treatments: Li^+^: 20% Pla: 14%	SAEs Li^+^: 9.4% Pla: 7.1% (“None of the serious AEs were believed to be related to study medication.”). Most common AEs - Li^+^: vomiting (45%), nausea (43%), headache (36%). - Pla: headache (32%), upper abdominal pain (32%), nausea and ↑ appetite (both 18%).	Li^+^ resulted more effective than Pla, with a generally acceptable adverse effect profile.	Brief duration of the study. Small sample. Impossibility of absolute certainty of the diagnosis in this cohort.
Patino *et al*., 2021 [27]	USA N = 109 in- or out-pts (10-17 y; M:44, F:65) with DSM-IV BD-I, mixed or manic episodes, YMRS > = 20 at BL, randomized: - Li^+^: N = 51 - Quet: N = 58	6-wk, randomized, DB, clinical trial. Primary outcome: BL-to-EP change in **YMRS.** Secondary outcomes: treatment response (50% or more ↓ from BL in YMRS score) and remission (YMRS score < = 12, CDRS-R < = 28 and CGI-BP-S < = 3). - Li^+^ dose:30 mg/kg (max starting dose 600 mg twice daily); target serum level: 1.0-1.2 mEq/L; - Quet dose:100 mg/d; target dose of 400-600 mg/d.	RR Li^+^ 49% Quet 72% For treatment response Quet superior to Li^+^ (p = 0.012); No differences in remission rates between groups were observed.	All reasons: Li^+^:41% Quet: 21% (p = 0.02) Attributable to treatments: Li^+^: 35% Quet: 19%	No SAEs. Most common AEs Li^+^: headaches (60.8%), nausea (39.2%), somnolence (27.5%), and tremor (27.5%); Quet: somnolence (63.8%), headaches (55.2%), tremor (36.2%), and dizziness (36.2%).	Quet was associated with a statistically significant greater rate of response and overall symptom reduction compared with Li^+^.	Small effect sizes. The difference between groups diminished with time, possibly related to the differences in DO rates.
Campbell *et al*., 1984 [28]	USA N = 61 inpts (5-13 yr; M:57, F:4) DSM-III diagnosis of CD- undersocialized, aggressive behavior: - Pla: N = 20 - HL: N = 20 - Li^+^: N = 21	4-wks, randomized, DB, Pla-controlled, trial (after 2 wks of Pla to screen out Pla-responder). Primary outcome: - Li^+^ dose:1.166 mg/day (range 500-2000 mg/day) - HL dose: optimal dose 2.95 mg/day (range 1-6 mg/day) - Psychometric assessment: **CPRS** (aggression), CGI, CTQ, PTQ, ABC.	RR (on behavior) by **CPRS** (aggression): - Pla: no factors showed significant improvement. - HL: less hyperactive (*P* = .001), less aggressive (*P* <.0001) and less hostile, (*P* = .007). - Li^+^: less hyperactive (*P* <.0001), less aggressive (*P* <.0001) less hostile (*P* = .001) and less unresponsive (*P* <.02). by CGI: HL did not differ from Li^+^, but the two drugs did differ from Pla. by CTQ, PTQ, ABC no significant results.	All reasons: not reported. Attributable to treatments: Li^+^:none. HL: not clear.	SAEs: none. Most common AEs: - HL: excessive sedation, acute dystonic reaction, and drooling; - Li^+^: stomachache, headache, and tremor of hands; - Pla: worsening of aggressiveness and excessive sedation.	Li^+^ and HL with significantly superior to Pla in decreasing behavioral symptoms. Although both medications were clinically effective, HL was associated more often with untoward effects than was Li^+^.	Short term study (4 wks). Small sample. An age-appropriate mood scale would be helpful to assess whether Li^+^ has a specific effect on mood explosiveness in these patients.
Campbell *et al*., 1995 [16]	USA N = 50 inpts (5-12 y; M:46, F:4) with CD, under socialized aggressive type (DSM III), behavioral profile of severe aggressiveness and explosiveness; failure to respond to previous outpatient treatments; normal intellectual functioning, randomized: - Pla: N = 25; - Li^+^: N = 25.	10-wk randomized, DB, Pla-controlled trial. - 2-wk Pla BL period, - 6 wk Li^+^ or Pla treatment, - 2 wk of Pla. - Li^+^ optimal daily dose of Li^+^ was 1,248 mg (600-1800 mg/die); serum level was 1.12 mEq/L. - Psychometric assessment: CPRS (aggression), GCJS, CGI, CTQ.	RR - CPRS: only the Aggression factor showed significant improvement with Li^+^ (*p* = .035); - GCJS: 68% Li^+^ markedly improved *vs.* 40% of Pla (*p* = .003); - CGI: global improvement for Li^+^(*p* = .044); no significance for severity of illness and efficacy index; - CTQ: Tension-Anxiety ↑ in Li^+^ group (less pts considered).	All reasons: not reported. Attributable to treatments: Li^+^:none.	SAEs/AEs. Untoward effects were associated both with Li^+^ and with Pla administration.	Li^+^ appears effective treatment for some severely aggressive children with conduct disorder.	Short duration of the study; no specific assessment for aggressive profile diagnosis so difficult to reproduce; no consideration of hyperactivity.
Rifkin *et al*., 1997 [29]	USA N = 33 inpts (12-17 y; M:14, F:19) with DSM-III CD: - Li^+^: N = 14 - Pla: N = 12	2-wk, randomized, single blind trial. Participants with ≥ 3 aggression episodes in 1 wk of hospitalization received single-blind Pla. Those who continued meeting eligibility criteria randomly assigned to Li^+^ or Pla. - Psychometric assessment: OAS (aggression). - Li^+^ dose: 600 mg/d; dose adjusted to maintain blood level 0.6-1.0 mM/L.	RR (OAS) 8.3% taking Pla and 21.4% taking Li^+^ reached remission at EP No differences among groups.	All reasons: 21.2%. Attributable to treatments: none.	SAEs: none. Most common AEs: There were more side effects in the Li^+^ group. Statistical significance in “autonomic side effects” and “distress attributable to symptoms”.	Li^+^ does not appear to benefit pts with CD in this trial.	Short duration of Li^+^ treatment (2 wk); exclusion of early Pla-responders.
Malone *et al*., 2000 [13]	USA N = 40 inpts (10-17 yr; M:33, F:7) with DSM-III-R CD and history of severe aggression, randomized: - Li^+^: N = 20; - Pla: N = 20.	6-wk, randomized, DB, Pla-controlled trial. - 2 wks Pla; - 4 wks trial Li^+^/Pla. Primary outcome/ psychometric assessment: OAS (aggression), GCJCS, CGI. - Li^+^ dose: Starting dose: 600 mg/die, ↑ if needed; serum level: 1.07 ± 0.19 mmoL; daily dose: 1425 mg/d.	RR: - by OAS: mean (SD) decrease from baseline in the Pla- group was –1.17 (4.15) compared with –2.40 (2.44) for the Li^+^ group (*p* = .04); - by GCJCS: 80% with Li^+^ *vs.* 30%) with Pla; - by CGI: 70% with Li^+^ *vs.* 20% with Pla. Lithium superior to placebo (*p* = 0.04)	All reasons: not reported. Attributable to treatments: none.	SAEs: not reported Most common AEs: Nausea, vomiting, and urinary frequency were more frequently associated with Li^+^ than Pla.	Li^+^ was more effective than placebo for reducing aggression in adolescent inpts and reaching overall clinical improvement.	Short duration, in a structured setting. No follow up.

**Abbreviations:** ABC, Timed Objective Rating Scale for Aggression; ACTeRS Attention Deficit Disorder-Hyperactivity Comprehensive Teacher Rating Scale; ADHD, attention-deficit/hyperactivity disorder; ADI, Adolescent Diagnostic Interview; AE, adverse event; Ap, antiepileptics; BD, bipolar disorder (type 1, BD-I; type 2, BD-II); BL, baseline; BMI, Body Mass Index; BPRS, Brief Psychiatric Rating Scale; CBZ, Carbamazepine; CD, conduct disorder; CDRS-R, Children’s Depression Rating Scale – Revised; CGAS, Children’s Global Assessment Scale; CGI, Clinical Global Impressions; CGI-I, Clinical Global Impressions – Improvement; CGI-BP, Clinical Global Impression-Bipolar Scale; CGI-BP-IM, Clinical Global Impressions for Bipolar Illness Improvement Mania; CGI-BP-S Bipolar Severity Scale; CGI-S, Clinical Global Impression – Severity scale; Chl, Chlorpromazine; CI, confidence Interval; CPRS, Children's Psychiatric Rating Scale; CPT Continuous Performance Test; C-SSRS, Columbia Suicide Severity Rating Scale; CTQ, Conners Teacher Questionnaire; DB, double-blind; DO, drop-out; DSM (-IV; -III;-III-R), The Diagnostic and Statistical Manual of Mental Disorder; DVPX, Divalproex sodium; Dz, diazepam; EP, endpoint; ET, early termination; F, female; FH, family history; GCJ, Global Clinical Judgments; GCJS, Global Clinical Judgments (Consensus) Scale; Ham-D, Hamilton Depression Rating Scale; HL, haloperidol; IGRS, Inpatient Global Rating Scale; KMRS, K-SADS (Kiddie Schedule for Affective Disorders and Schizophrenia) Mania Rating Scale; K-SADS-E, Kiddie-Schedule for Affective Disorders and Schizophrenia for School Age Children; K-PAL, Paired Associated Learning paradigm; Li^+^, lithium; LOCF, last-observation-carried-forward; LSES, Lithium Side Effects Scale; M, male; MDD, Major Depressive Disorder; mEq/L, milliequivalents per liter; mM/L, millimoles per liter; mo, month(s); MPH, methylphenidate; MRS, Mania Rating Scale; NR, non-responder; NS, not significant; OAS, Overt Aggression Scale; p, p-value; pt(s), patient(s) (inpts, inpatients; outpts, outpatients); PANSS, Positive And Negative Syndrome Scale; PGBI-10M, Parent General Behavior Inventory-10 Item Mania Scale; PFT, Rosenzwig’s Picture Frustration Test; Pla, placebo; POMS, Profile of Mood States; Pro, promethazine; PT, partial responder; PTQ, Conners Parent-Teacher Questionnaire; Quet, quetiapine; Risp, risperidone; RR, response, rates; SAE, side adverse effect; SAPS, Scale for the Assessment of Positive Symptoms; SDD, substance dependence disorder; SMD, Severe Mood Dysregulation; SUD, substance use disorder; TDZ, thioridazide; Thi, thiothixene; Trim, trimethozine; US, united state; wk, week(s); mean; yr, year(s).

**Table 2:** Demographic and clinical characteristics of the included samples.

**Study**	**Mean Age (Years)**	**Sex (%)**	**Duration of Illness (Years ± SD)**	**Age at Onset (Years ± SD)**	**CD, ADHD, ODD, and DMDD Comorbidity**	**Other Farm Exposure**	**Recruitment Setting**
**Bipolar Disorder**							
Geller *et al*., 1998	Tot: 16.3 Li^+^: 16 Pla: 16.6	Tot: M 64, F 36 Li^+^: M 61.5, F 38.5 Pla: M 66.7, F 33.7	At least 2 mo	Not reported	Not reported	Unspecified	Outpts
Kowatch *et al*., 2000	Tot: 11.4	M 62 F 28	Tot: 4.6 ± 2.8	Tot: 7.1 ± 3.4	CD:7% ADHD: 71% ODD: 38%	79% prior psychotropic medications with a 2wk wash-out period	Outpts
Kafantaris *et al*. 2004	Tot: 15.16 Li^+^: 15.08 Pla: 15.23	Tot: M 50, F 50 Li^+^: M 52.6, F 47.4 Pla: M 47.6, F 52.4	Not reported	Not reported	Any comorbid diagnosis: Li^+^: 73.9% Pla:85.7%	25% had been treated initially with an adjunctive antipsychotic medication. These subjects were equally distributed between Li^+^ (26.3%) and Pla (23.8%)	Inpts
Pavuluri *et al*., 2004	Tot: 12.1 Li^+^/Risp: 12.06 DVPX/Risp: 12.10	Li^+^/Risp: M 65, F 35 DVPX/Risp: M 65, F 35	Not reported	Not reported	ADHD: Li^+^/Risp: 82.4% DVPX/Risp: 75% ODD: Li^+^/Risp: 58.8% DVPX/Risp: 25%	Psychostimulants clonidine, trazodone, benztropine allowed	Outpts
Findling *et al*., 2005	Tot: 10.7 Li^+^: 10.3 DVPX: 11.2	Tot: M 65, F 35 Li^+^: M 70, F 30 DVPX: M 60, F 40	Weeks: Tot: 149.7 ± 115.4 Li^+^: 162.6 ± 117.9 DVPX: 136.8 ± 113.4	Tot: 7.3 ± 4.1 Li: 6.7 ± 4.0 DVPX: 8.0 ± 4.1	ADHD: 58.3%	Pts were tapered off prior antipsychotics and antidepressants during phase 1. Stable psychostimulants allowed during phase 2 (58.3%)	Outpts
Geller *et al*., 2012 (Team Study)	Tot: 10.1 Li^+^: 9.7 Risp: 11 DVPX: 9.7	Tot: M 49.8, F 50.2 Li^+^: M 58.9, F 41.1 Risp: M 52.8, F 47.2 DVPX: M 44, F 56	4.8 ± 2.6	5.0 ± 2.7	CD:15.8% ADHD: 92.8% ODD: 90% DMDD: 98.6%	Antimanic medication naive subjects stable psychostimulant medication: 32.3%	Outpts
Findling *et al*., 2015 (CoLT 2)	Li^+^: 11.5 Pla: 11.2	Li^+^: M 41.5, F 58.5 Pla: M 53.6, F 46.4	Not reported	Not reported	ADHD: Li^+^: 64.2%; Pla: 64.3% DMDD: Li^+^: 20.8%; Pla: 21.4%	Li^+^ naïve and willing to undergo a washout period for prior medication Psychostimulants allowed after 4 weeks of double-blind therapy	Outpts
Patino *et al*., 2021	Li^+^: 15.0 Quet: 14.2	Li^+^: M 37,F 63 Quet: 43, F 57	Li:^+^ 1.1 ± 1.2 Quet:1.3 ± 1.5	Li^+^: 13.8 ± 2.1 Que: 13.0 ± 2.2	-	Prior medication exposure: Li^+^ 40% Quet 43%	Inpts and outpts
**Conduct Disorder**							
Campbell *et al*., 1984	Tot: 8.97	M 93.4 F 6.6	Not reported	Not reported	Not reported; psychosis excluded	No concurrent medication	Inpts
Campbell *et al*., 1995	Tot: 9.4 ± 1.8	M 92 F 8	Not reported	Not reported	-	Failure to respond to previous pharmacotherapy as an inclusion criteria.	Inpts
Rifkin *et al*., 1997	Tot: 15.15 ± 1.48	M 43 F 57	Not reported	Not reported	Not reported; mood disorders excluded	Unspecified	Inpts
Malone *et al*., 2000	Tot: 12.5 Li^+^: 12.3 Pla: 12.6	Li^+^: M 85, F15 Pla: M 80, F 20	Not reported	Not reported	Not reported mood disorders excluded	Pts who received psychoactive medication within 2 weeks of the study or had a previous Li^+^ trial were excluded No concurrent medication	Inpts

**Abbreviations:** ADHD, attention-deficit/hyperactivity disorder; CD, conduct disorder; DMDD, disruptive mood dysregulation disorder; DVPX, Divalproex sodium; F, female; Li^+^, lithium; M, male; mo, month(s); ODD, oppositional defiant disorder; pt(s), patient(s) (inpts, inpatients; outpts, outpatients); Pla, placebo; Quet, quetiapine; Risp, risperidone; SD, standard deviation; Tot, total, wk, week(s); yr, year(s).

## LIST OF ABBREVIATIONS

ADHD: Attention Deficit Hyperactivity Disorder
BD: Bipolar Disorder
CD: Conduct Disorder
DMDD: Disruptive Mood Dysregulation Disorder
ODD: Oppositional Defiant Disorder
RoB: Risk-of-Bias
SAEs: Severe Adverse Events

## STANDARDS OF REPORTING

PRISMA guidelines and methodology were followed.
