# Supplementary material for: Use of Lithium in Pediatric Bipolar Disorders and Externalizing Childhood-related Disorders: A Systematic Review of Randomized Controlled Trials
Source: Curr Neuropharmacol. 2023 May 12;21(6):1329–42. doi: 10.2174/1570159X21666230126153105 (PMC10324336; doi:10.2174/1570159X21666230126153105)
Supplement: Supplementary file 1 [file CN-21-1329_SD1.pdf]

Supplementary Material

Use of Lithium in Pediatric Bipolar Disorders and Externalizing Childhood-related Disorders: A Systematic Review of Randomized Controlled Trials

Delfina Janiri[1,2,*], Lorenzo Moccia[1,2], Silvia Montanari[1], Valentina Zani[1], Claudia Prinari[1], Laura Monti[1,3], Daniela Chieffo[1,3], Marianna Mazza[1,2], Alessio Simonetti[1,2,4,5], Georgios D. Kotzalidis[1,2,4,6] and Luigi Janiri[1,2]

[1]Department of Geriatrics, Institute of Psychiatry and Psychology, Neuroscience and Orthopedics, Catholic University of the Sacred Heart, Rome, Largo Francesco Vito 1, 00168, Rome, Italy; [2]Fondazione Policlinico Universitario Agostino Gemelli IRCCS, Largo Agostino Gemelli 1, 00168, Rome, Italy; [3]UOS Clinical Psychology, Clinical Government, Fondazione Policlinico Universitario Agostino Gemelli IRCCS, Largo Agostino Gemelli 1, 00168, Rome, Italy; [4]Centro Lucio Bini, Via Crescenzio 42, 00193, Rome, Italy; [5]Menninger Department of Psychiatry and Behavioral Sciences, Baylor College of Medicine, 1 Baylor Plaza, 77030, Houston, TX, USA; [6]NESMOS Department, University of Rome La Sapienza, Faculty of Medicine and Psychology, Sant'Andrea University Hospital, Via di Grottarossa, 1035-1039, 00189, Rome, Italy

eMethods

I. Search strategy
II. Article eligibility
III. Data extraction

eResults

I. Results
II. Prisma Flow chart
III. Supplementary Table 1

eReferences

1875-6190/23 $65.00+.00 **© 2023 Bentham Science Publishers**

Search Strategy

We conducted a systematic literature search in accordance with the Preferred Reporting Items for Systematic Reviews and meta-analyses criteria (http://www.prisma-statement.org/) (eFigure 2) to identify longitudinal controlled clinical trials using lithium in pediatric bipolar disorder, conduct disorder, attention deficit hyperactivity disorder (ADHD), oppositional defiant disorder and disruptive mood dysregulation disorder published from any time to June 4, 2022 in PubMed (http://www.pubmed.org), Web of Science (https://apps.webofknowledge.com) and Google Scholar (https://scholar.google.de). The search keywords were: “lithium” “children”, “pediatric”, “youth”, “adolescents”, “bipolar disorder”, “conduct disorder”, “attention deficit hyperactivity disorder (ADHD)”, “oppositional defiant disorder”, “disruptive mood dysregulation disorder” and their various combinations and permutations.

PRISMA Flowchart from database search and strategy used is shown in the Supplementary Results section.

Article Eligibility

We included articles that: (a) investigated the effect of lithium; (b) were randomized controlled trial; (c) compared lithium with placebo or an alternative active drug; (d) examined pediatric populations; (e); studied patients with bipolar disorder, conduct disorder, attention deficit hyperactivity disorder, oppositional defiant disorder, or disruptive mood dysregulation disorder diagnoses (f) used the diagnostic criteria of the Diagnostic and Statistical Manual of Mental Disorders (DSM) or the International Statistical Classification of Diseases and Related Health Problems (ICD) (e) reported efficacy outcomes.

Exclusion criteria were: (a) reviews and meta-analyses (although we used their reference lists to seek possible additional eligible studies that could have eluded our search strategy); (b) studies not including pediatric population; (c) studies including multiple diagnoses without providing data for BD,CD, ODD, DMDD separately; (d) studies without efficacy outcomes; (g) samples with serious concomitant medical illnesses, diagnosed intellectual disability, or brain injury; (h) studies with overlapping samples.

We mostly included randomized blind controlled trials but choosing only this type of studies would have created a selection bias. Therefore, according to a previous study (1), we maintained the controlled and the randomization criterion, but no blindness restriction was imposed. Regarding the samples included in the studies comorbidity was accepted, but not multiple diagnoses (i.e., heterogeneous diagnostic samples). When articles reported results from overlapping samples, we included the article reporting primary outcomes and with the largest sample size. No language limitations were imposed.

Lithium could be administered by any method at any dose falling within or close to the therapeutic range (0.4-1.2 mmol/l); there was no stringent requirement for a particular lithium dosing strategy.

Five authors (DJ, SM, VZ, CP, GDK) separately inspected all the titles and abstracts of articles collected from the electronic database searches. After obtaining the full-text version of the relevant articles, the reviewers individually re-evaluated them. Eligibility was established with consensus among five authors (DJ, SM, VZ, CP, GDK) through Delphi rounds carried out with online meetings. Three were sufficient to reach consensus (75% agreement).

Primary and secondary outcomes

The primary outcome of our study was to evaluate efficacy of lithium in paediatric age defined by increase/decrease in scores on any validated rating scale from baseline and endpoints, between individuals treated with lithium and individuals treated with placebo or other pharmacological means. As secondary outcomes we also considered tolerability and acceptability. Tolerability was defined as difference in serious adverse events between the lithium and the control group; Acceptability as difference in discontinuation rates for any reason between lithium and the control group.

Data extraction

Specific data of the eligible full-version articles were carefully extracted and filled into the developed extraction form. The extracted outcomes, when available from each eligible study, consisted of the following:

(i)Data on efficacy, tolerability and acceptability. For the efficacy outcome, response rates were reported or reported if available; (ii) demographic and clinical characteristics of included patients (mean age, sex ratio, diagnosis, duration of illness, illness onset, prior pharmacological treatment, recruitment setting); (iii) Design of the study including information on blindness vi) conclusions and limitations (when reported in the original study).

Results

The above-mentioned search produced 2550 records on PubMed, Web of Science (limiting to pediatrics), and Google Scholar.

At end of the eligibility process we included 12 independent trials, for a total of 857 patients. All the included studies were randomized controlled trials, written in English. Included studies spanned from 1984 to 2021. The results of our search are shown as a *PRISMA* flowchart in Supplementary Figure 1 with the reasons of exclusion.

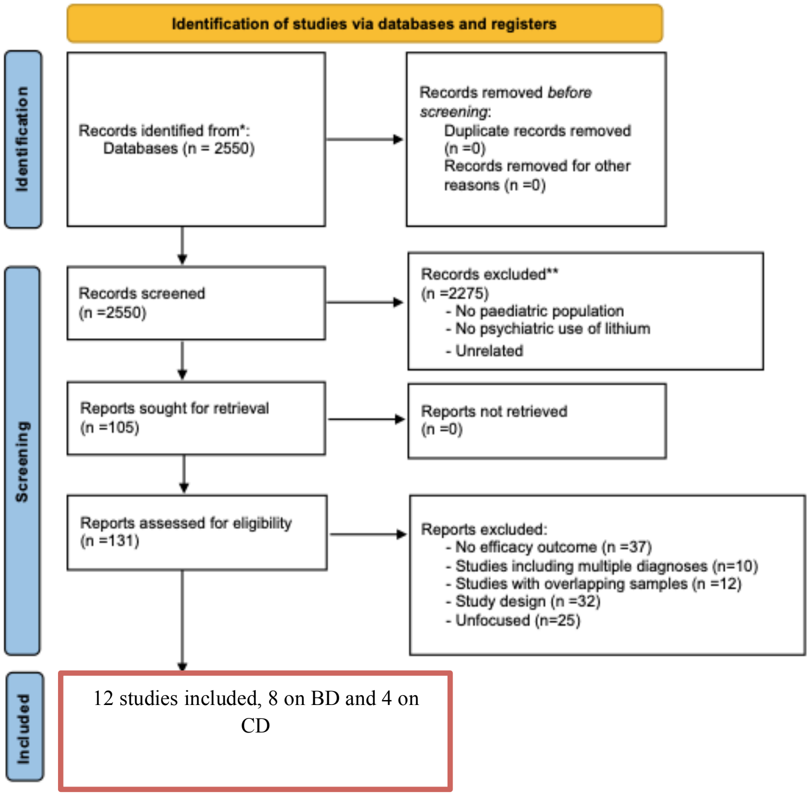

Supplementary Figure 1: PRISMA flow diagram of included studies.

For more information, visit: http://www.prisma-statement.org/

Two of the included studies were parts of the Treatment of Early Age Mania Study (TEAM) and of The Collaborative Lithium Trials (CoLT). Regarding the TEAM study, the study that was considered as the primary study was Geller et al. (2), where drug-naïve patients (for anti-manic drugs) were randomized to three parallel groups (lithium, divalproex, risperidone); patients who did not respond to this phase of the study together with those who were not-drug naïve at the beginning, were then included in the next phase of the study (cross taper/add on) described in Walkup et al. (3). This study was therefore considered an additional TEAM because it is not possible to determine which patients overlap with those of the Geller study (2). The other two studies included as additional are Salpekar et al. (4) and Vitiello et al. (5), both post hoc analyses of the same patients of Geller et al., but investigating other specific areas (the first reduction of suicidality, the second treatment moderators and predictors of outcome). The additional TEAM studies are reported in Supplementary Table 1. Regarding the CoLT study, the study that was considered as the primary study was Findling et al. (6), a randomized, double blind controlled trial. Two other papers that were parts of the CoLT study were excluded because they did not compare lithium with placebo or an alternative active drug (7,8). Another study reported data on maintenance and discontinuation due to mood symptoms but considering a smaller sample size (9). The additional CoLT studies are summarized in Supplementary Table 1.

Supplementary Table 1. Summary of TEAM-related and CoLT-related additional studies.

Study	Patients	Design	Results	Acceptability/ Discontinuations	Tolerability/Adverse Effects	Conclusion	Limitations/Observations
BD							
Additional TEAM*<br><br>Vitiello et al., 2012 [5]	Same samlple of the primary TEAM study:<br>N=279 outpts (6-15 y; M:139,F:140) with DSM-IV BD-I, mixed or manic episode for at least 4 wks preceding BL, with CGAS ≤ 60, randomized:<br><br>- Li^+: N=90 (age x̄=9.7), M:[illegible], F:47;<br>- Risp: N=89 (age x̄=10) M:[illegible], F:47;<br>- DVPX: N=100 (age x̄=9.7) M:4[illegible], F: 56.	8-wk, randomized, single blind trial.<br><br>Primary outcome: treatment moderators and predictors of outcome.	RR by<br>- Site differences:<br>Risp vs Li^+ :2.6 to 8.3 (p=0.22-0.037);<br>Risp vs DVPX: 1.3 to 10.5 (not significant).<br>Li^+ vs DVPX: no difference.<br>- ADHD:<br>Risp vs Li^+ :2.1 (p=0.44)<br>- obesity: T<br>Risp vs Li^+ : 1.1 vs 2.3 in nonobese ones (p=0.664 - 0.0001)<br>- no moderating effects of ODD.	Same primary TEAM study.	Same primary TEAM study.	Risp was more effective than Li^+ or DVPX across the demographics and clinical characteristics of the sample, but the magnitude of its effect was influenced by site-related characteristics and presence of ADHD.	Same T primary TEAM study.:<br>No valid diagnostic biological measure for childhood BP disorder. Uncertain validity of preschool diagnoses. Too few nonpsychotic subjects for meaningful analyses of this subgroup.
Additional TEAM*<br><br>Walkup et al., 2015 [3]	Pts from the primary TEAM study, PT and NR, at BL + after 8-wks of treatments.<br>N=65 PT randomized to add-on with one of the other treatments:<br>- N=15 Risp add-on<br>- N=30 Li^+ add-on<br>- N=20 DVPX add-on.<br>N=89 NR switched to one of the other treatments:<br>- N=21 to Risp<br>- N=39 to Li^+<br>- N=29 to DVPX.	8-wk, randomized, unblinded, add-on or cross-taper strategy study.<br>Primary outcome: clinical response by the 7-point CGI-BP-IM.	PT RR:<br>Risp add-on (53.3%) higher than DVPX add-on (0%) and Li^+ (26.7%).<br><br>NR RR:<br>switch to Risp (47.6%) higher than DVPX (17.2%) and Li^+ (12.8%).	All reasons:<br>Li^+ : 50%<br>Risp: 13%<br>DVPX: 60%<br><br>Attributable to treatments: not reported.	SAEs:<br>PT:4.6%<br>NR: 6.7%<br>(no medication distribution reported)<br><br>Most common AEs:<br>-Li^+ :difficulty arousing in AM, gastrointestinal symptoms, weight loss, enuresis, thrist. DVPX: drowsiness and difficulty rousing in the morning.<br>-Risp: weight gain, difficulty concentrating, nasal congestion, dry mouth, enuresis, excessive thirst.<br><br>All three medications associated with weight gain.	Risp was more useful than Li^+ and DVPX in reducing mania symptoms in children when added to a mood stabilizer in pts with a partial response or when used as monotherapy in those who failed an initial antimanic medication.	No Pla-controlled. Unblinded design. Small sample sizes for each group.
Additional TEAM*<br><br>Salpekar et al., 2015 [4]	Same sample of the primary TEAM study:<br>N=279 outpts (6-15 y; M:139,F:140) with DSM-IV BD-I, mixed or manic episode for at least 4 wks preceding BL, with CGAS ≤ 60, randomized:<br><br>- Li^+: N=90 (age x̄=9.7), M:[illegible], F:47;<br>- Risp: N=89 (age x̄=10) M:[illegible], F:47;<br>- DVPX: N=100 (age x̄=9.7) M:4[illegible], F: 56.	8-wk, randomized, single blind trial.<br>Primary outcome: to assess the efficacy of mood-stabilizing medications for depression and suicidality.<br><br>Psychometric assessment: CGI-BP-ID; CDRS-R; suicidality status (item 13 of CDRS-R).	RR (CGI-BP-ID) Risp: 60.7% Li^+:42.2% DVPX: 35.0%.<br><br>CDRS-R showed early improvement for Risp compared to the other groups, but didn't differ in degree.<br><br>item-13: no significant difference.	Same primary TEAM study:<br><br>All reasons:<br>Li^+ : 32.2%<br>Risp: 15.7%<br>DVPX: 26.0%	Same primary TEAM study	Depressive symptoms improved with all 3 treatments; Risp yielded more rapid improvement than Li^+ or DVPX. Suicidality was infrequent, and there was no overall effect of treatment on suicidality ratings.	Secondary review of data. Missing data because of DO. Sample size.
Additional CoLT**<br><br>ColT1 study (phase I)<br><br>Findling et al., 2011 [7]	N=60 pts (7-17 yr) with DSM-IV BD-I, mixed or manic episodes, were eligible in three dosing arms with Li^+.<br>- Arm I (N=20): Li^+ 300mg x2/day, ↑ by 300mg/day if needed;	8-wk, randomized open, dose-based, (phase I) trial.<br><br><br>- Li^+ dose x̄ daily dose: 1[illegible]0mg, x̄ serum concentration: 1.05	RR (by YMRS) 61.7% had a ≥ 50% improvement 58,3% achieved response.	All reasons:<br>Arm I: 25%<br>Arm II: 38%<br>Arm III: 37%<br><br>Attributable to treatments:<br>Arm I: 15%<br>Arm II: 10%<br>Arm III: 11%	SAEs: 10%<br>Most common AEs: gastrointestinal disorders, dizziness, headache, tremor, somnolence, fatigue , thirst, pollakiuria.	Li^+ was associated with significant amelioration in children with BP-I. The obtained dosing paradigm is: starting dose of 300mg of Li^+ x3/day,	Open, uncontrolled design. Brevity and relatively small sample size. Outpts trial, so the Li^+ levels may not have been fully accurate. No information about possible use of Li^+ as maintenance treatment. High DO rates.

	- Arm II (N=21): Li^+ 300mg x3/day, ↑ by 300mg/day if needed;<br>- Arm III (N=19): Li^+ 300mg x3/day, ↑ by 300mg/day, if needed, after mid-wk telephone interview.	mEq/l.<br>- Psychometric assessment: YMRS, CDRS-R, CGI-S, CGI-I, CGAS and others.				followed by 300mg weekly ↑, until an improvement is reached.	
Additional CoLT**<br><br>CoLT1 study (phase II)<br><br>Findling et al., 2013 [8]	N=41 pts (7-17 yr) with DSM-IV BD-I, mixed or manic episodes, treated for 8 wks (phase I) with Li^+ and reached 25% reduction in YMRS and CGI-I score ≤3:<br>-Same Li^+ dose used at the end of phase I at the onset of this trial;<br>- Up to 2 adjunctive medications for residual symptoms / comorbid conditions.	16-wk, open label, long term effectiveness (phase II) trial.<br><br>- Li^+ dose $\bar{x}$ dose: 1470.7 mg/d; serum level: 1.0 mEq/L.<br><br>- Psychometric assessment: YMRS, CDRS-R, CGI-S, CGI-I, CGAS and others.	RR<br>- ≥ 50% ↓ in YMRS score: 73.2%<br>- YMRS (50%↓) +CGI: 68,3% (criteria for response);<br>- YMRS<12 + CGI: 53.7% (in remission).	All reasons: 49%<br><br>Attributable to treatments: 27%	No SAEs.<br>Most common AEs: gastrointestinal disorders, headache, tremor, enuresis, thirst.	Li^+may be a safe and effective longer-term treatment for patients who respond to acute treatment. PR did not experience substantial symptom improvement during the continuation phase.	Open, uncontrolled design. Small sample size. Brevity.
Additional CoLT**<br><br>Findling et al., 2019 (pts from CoLT1 e CoLT2)[9]	N=31 pts (7-17 y) with criteria for BD-I, mixed or manic episode (YMRS<10; CDRS-R<35):<br>N: 21 pts from CoLT1<br>N: 10 pts from CoLT2<br>Randomized:<br><br>- Li^+: N=17 (age, [illegible]<br>- Pla: N=14 (age [illegible]	4-wk, randomized, DB, Pla-controlled discontinuation study (after 24-wk post-acute open-label treatment trial).<br>PO: relative risk of study discontinuation for any reason.<br><br>- Li^+ dose: BL ≥0.6 mEq/L; 0.8 and 1.2 mEq/L.<br>- Psychometric assessment: CGI-I; CGI-S; CGAS; YMRS; CDRS-R; PGBI-10M.	RR<br>Discontinuation due to mood symptoms occurred in 29% of participants treated with Li^+ compared to 71% with Pla (p=0.013).	All reasons:<br>Li^+ :35%<br>Pla: 86%	SAEs:<br>- Li^+:5.8%(1pt)<br>-Pla:none<br><br>Most common AEs: - Li^+: headache (35%), upper abdominal pain (29%), enuresis (24%), vomiting (24%), initial insomnia (18%), upper respiratory tract infection (18%), ↓ appetite (12%) and nasopharyngitis (12%).<br>- Pla: headache (29%), initial insomnia (14%) and ↓ appetite (14%).	This trial supports the role of Li^+ as a maintenance treatment in pediatric pts with BD.	Small sample size and trial duration.

Legend: * Regarding the Treatment of Early Age Mania Study (TEAM) study, the study that was considered as the primary study was Geller et al. Table 1 in the main). ** Regarding the The Collaborative Lithium Trials (CoLT) study, he study that was considered as the primary study was Findling et al. (Table 1 in the main).

Abbreviations: BD, bipolar disorder (type 1, BD-I; type 2, BD-II); MDD, Major Depressive Disorder; CD, conduct disorder; DD, depressive disorder; ADHD, Attention Deficit Hyperactivity Disorder; SDD, substance dependence disorder; SUD, substance use disorder; ODD, oppositional defiant disorder; DSM (-IV; -III;-III-R), The Diagnostic and Statistical Manual of Mental Disorder; EUCD, emotionally unstable character disorder; ADD, attention deficit disorder; DB, double-blind; DO, drop-out; BL, baseline; EP, endpoint; Li^+, lithium (-C, carbonate; -S, sulfate); VAL, divalproex sodium; RISP, rispderidone; HL, haloperidol; Carb, carbamazepine; MPH methylphenidate; nl, neurolepltic; mEq/L, milliequivalents per liter; mM/L, millimoles per liter; mo, month(s); Pla, placebo; pt(s), patient(s) (inpts, inpatients; outpts, outpatient[illegible]
↑, increase; ↓, decrease; p, p-value; EP, evoked potentials; AE, adverse event; SAE, side adverse effect; AD, anxiety disorder; NR, non responder; PT, partial responder; TR, Simple Reaction Time; RR, response rates; PMS, Porteus Maze score; MFFT, Matching Familiar Figure Test; K-SADS (Kiddie Schedule for Affective Disorders and Schizophrenia) Mania Rating Scale; K-SADS-E Kiddie-Schedule for Affective Disorders and Schizophrenia for School Age Children; CDRS-R, Children's Depression Rating Scale-Revised; CGI-BP Clinical Global Impression-Bipolar Scale; CGI-I, Clinical Global Impressions – Improvement; CGI-S, Clinical Global Impression – Severity scale; YMRS, Young Mania Rating Scale; MRS, Mania Rating Scale; CGAS, Children's Global Assessment Scale.

eReferences

1. Duffy A, Heffer N, Goodday SM, Weir A, Patten S, Malhi GS, et al. Efficacy and tolerability of lithium for the treatment of acute mania in children with bipolar disorder: A systematic review: A report from the ISBD-IGSLi joint task force on lithium treatment. Bipolar Disord. 2018;20(7):583–93.

2. Geller B, Luby JL, Joshi P, Wagner KD, Emslie G, Walkup JT, et al. A randomized controlled trial of risperidone, lithium, or divalproex sodium for initial treatment of bipolar I disorder, manic or mixed phase, in children and adolescents. Arch Gen Psychiatry [Internet]. 2012 May 1;69(5):515–28. Available from: http://archpsyc.jamanetwork.com/article.aspx?doi=10.1001/archgenpsychiatry.2011.1508

3. Walkup JT, Wagner KD, Miller L, Yenokyan G, Luby JL, Joshi PT, et al. Treatment of Early-Age Mania: Outcomes for Partial and Nonresponders to Initial Treatment. J Am Acad Child Adolesc Psychiatry [Internet]. 2015 Dec;54(12):1008–19. Available from: http://dx.doi.org/10.1016/j.jaac.2015.09.015

4. Salpekar JA, Joshi PT, Axelson DA, Reinblatt SP, Yenokyan G, Sanyal A, et al. Depression and Suicidality Outcomes in the Treatment of Early Age Mania Study. J Am Acad Child Adolesc Psychiatry [Internet]. 2015 Dec;54(12):999-1007.e4. Available from: https://linkinghub.elsevier.com/retrieve/pii/S0890856715006474

5. Vitiello B, Riddle MA, Yenokyan G, Axelson DA, Wagner KD, Joshi P, et al. Treatment Moderators and Predictors of Outcome in the Treatment of Early Age Mania (TEAM) Study. J Am Acad Child Adolesc Psychiatry [Internet]. 2012 Sep;51(9):867–78. Available from: https://www.ncbi.nlm.nih.gov/pmc/articles/PMC3624763/pdf/nihms412728.pdf

6. Findling RL, Robb A, McNamara NK, Pavuluri MN, Kafantaris V, Scheffer R, et al. Lithium in the Acute Treatment of Bipolar I Disorder: A Double-Blind, Placebo-Controlled Study. Pediatrics [Internet]. 2015 Nov 1;136(5):885–94. Available from: http://www.ncbi.nlm.nih.gov/pubmed/26459650

7. Findling RL, Kafantaris V, Pavuluri M, McNamara NK, McClellan J, Frazier JA, et al. Dosing Strategies for Lithium Monotherapy in Children and Adolescents with Bipolar I Disorder. J Child Adolesc Psychopharmacol [Internet]. 2011 Jun;21(3):195–205. Available from: http://www.liebertpub.com/doi/10.1089/cap.2010.0084

8. Findling RL, Kafantaris V, Pavuluri M, McNamara NK, Frazier JA, Sikich L, et al. Post-Acute Effectiveness of Lithium in Pediatric Bipolar I Disorder. J Child Adolesc Psychopharmacol [Internet]. 2013 Mar;23(2):80–90. Available from: http://www.liebertpub.com/doi/10.1089/cap.2012.0063

9. Findling RL, McNamara NK, Pavuluri M, Frazier JA, Rynn M, Scheffer R, et al. Lithium for the Maintenance Treatment of Bipolar I Disorder: A Double-Blind, Placebo-Controlled Discontinuation Study. J Am Acad Child Adolesc Psychiatry [Internet]. 2019 Feb;58(2):287-296.e4. Available from: https://linkinghub.elsevier.com/retrieve/pii/S089085671831983X
